# The Cardiac Muscle

**Published:** 1904-02-27

**Authors:** 


### THE CARDIAC MUSCLE.

It is perhaps because their clinical features are not so easily demonstrated that diseases of the myocardium are by most teachers and text-books treated with less detail and emphasis than those due to lesions of the valves. This is at least to be regretted, for the actual termination of a case of valvular heart disease is due to failure of the cardiac muscle to overcome the actual strain thrown upon it by the valvular lesion. Drugs and other forms of treatment are likely to have but little effect upon the valves themselves once they have become affected. It is the heart muscle that requires the physician's care. Nor is it in valvular disease only that the condition of the myocardium is of importance; in atheroma of the coronary arteries and in other conditions both local and general the myocardium is found to undergo well marked changes. Dr. John M. Cowan[1] deals in a lucid manner with the pathological aspects of the matter. He states that in a heart which is notably larger than usual, the average size of the cells is always greater than normal, the increase being especially evident in the transverse diameter; he suggests that it may be in this way, and not by a new formation of muscle cells that the cardiac power is increased. Conversely, in the atrophied hearts found post-mortem after wasting diseases such as phthisis and diabetes, the muscle cells are markedly smaller than normal. In addition to illnesses causing general emaciation, local causes also may produce atrophy by interfering with the proper blood supply, such as atheroma of the coronary arteries and narrowing of their orifices by atheroma of the aorta.

In acute infectious diseases the change known as cloudy swelling affects the cardiac muscle as it does the cells of other organs. In this condition the cells are filled with minute granules, which, in the fresh state, obscure all the proper structures. In stained specimens these granules are usually not apparent. If a toxæmia has existed for any length of time, cloudy swelling of the heart muscle is almost always present, and of necessity it materially weakens the force of the cardiac systole; consequently pneumonia proves fatal most frequently in elderly people, or in those in whom the heart is working at nearly its full power owing to some chronic affection, such as old valvular incompetence.

Fatty degeneration of the myocardium is not uncommon. There are two varieties met with, of different etiology. In one set of cases the fatty degeneration is patchy in its distribution producing the "thrush-breast" appearance. This condition is best seen in anæmic cases. Microscopical sections stained with osmic acid show that in the immediate neighbourhood of the arterioles, the muscle-cells are free from fatty degeneration, while some distance away, almost the whole of every cell shows evidence of degenerative changes. So that it is in those parts where the blood supply is the less free where fatty degeneration is most apt to occur In the second variety the distribution of the fatty change is not patchy, but general, and its origin is different. The muscle cells near the arterioles are equally affected with those further away. The cause is a toxic one, the condition being observed after pericarditis, in which case that portion of the myocardium which is adjacent to the pericardium is most markedly affected, or it may be due to toxins circulating in the blood. Thus it occurs in renal insufficiency, long continued sepsis, and alcoholism. Experimentally, this kind of fatty degeneration can be readily produced in rabbits by the subcutaneous injection of phosphorus or chloroform, while the continued administration of arsenic produces the same effect. There is a third form of fatty heart, namely, that in which there is fatty infiltration. In these cases the connective tissue of the heart contains large quantities of fat, and the muscle cells in the vicinity of this interstitial fat are themselves fatty. So that the heart movements are impeded mainly by the presence of the masses of fat, but partly also by fatty degeneration of some of the cells. Usually this condition accompanies excessive development of fat throughout the body generally, although exceptions to this rule are occasionally found.

Fibrosis of the heart may occur in a single patch or as a general condition. It is usually the result of alterations in the coronary circulation, whether caused by lesions of the arteries themselves — atheroma, thrombosis, embolism—or of their orifices above the sigmoid valves—aortic atheroma, etc. The local or general malnutrition of the heart caused by such lesions leads to atrophy of the muscle cells and their replacement by fibrous tissue. The distribution of the fibrosis is similar to that of the fat in anæmic fatty degenerations. In the latter case the cause is an improper blood supply, in the former an insufficient blood supply. There is another form of fibrosis of the heart which follows acute infections such as pneumonia. In the acute stage small scattered infiltrations of mononuclear cells occur with destruction of the muscle cells in their immediate vicinity, and as recovery takes place little patches of fibrous tissue are left.

Dr. Cowan states that pericardial inflammations have little effect in producing fibrous tissues in the heart, and this he believes to be explained by the fact that the flow in the veins and lymphatics of the heart muscle is towards the pericardium and not in the reverse direction.

[1] Edin. Med. Jour., Feb. 1904.

